# Effect of post‐COVID‐19 condition on sleep: A case report

**DOI:** 10.1002/ccr3.7149

**Published:** 2023-03-27

**Authors:** Asma Salameh Albtoosh, Sajeda Ghassan Matar, Shatha Nizar Bishtawi, Alaa Ahmed Elshanbary, Lara Ibrahim Ramadan, Andrew Bradbeer, Elfatih A. Hasabo, Iman A. Basheti

**Affiliations:** ^1^ Internal Medicine Department University of Jordan Hospital Amman Jordan; ^2^ Faculty of Pharmacy Applied Science Private University Amman Jordan; ^3^ General Surgery Department University of Jordan Hospital Amman Jordan; ^4^ Faculty of Medicine Alexandria University Alexandria Egypt; ^5^ Western District Health Services Hamilton Victoria Australia; ^6^ Faculty of Medicine University of Khartoum Khartoum Sudan; ^7^ Sudan Analytics Research Group Khartoum Sudan; ^8^ Faculty of Pharmacy Applied Science Private University 11931 Amman Jordan; ^9^ Faculty of Pharmacy The University of Sydney 2006 Sydney New South Wales Australia

**Keywords:** COVID‐19, insomnia, pharmacist, sleep

## Abstract

Post‐COVID‐19 condition affects patients on various aspects. This 41‐year‐old female presented to the outpatient clinic complaining of severe insomnia characterized by inconsistent 2 h of sleep per day despite taking sleep aid pills after being infected with COVID‐19 and persisting for 6 months after recovery.

## 1 INTRODUCTION

Post‐COVID‐19 condition is characterized by many symptoms starting from fatigue and dyspnea, while more persistent symptoms can include smell and taste dysfunction, joint pain, palpitations, gastrointestinal (GI) issues, and headaches.^1^ The COVID‐19 pandemic was proved to affect mental and cognitive health, especially in affecting anxiety and depression among patients.^2^ In addition to affecting sleep quality, many studies assessed the prevalence of insomnia during the pandemic, which ranges between 37.6% and 23.8% based on previous studies.^3^, ^4^ When it comes to the post‐COVID‐19 condition, a study conducted by Xu et al. indicates that rates of insomnia were 26.4% among COVID‐19 survivors 2 weeks after discharge,^5^ although there are not enough data available yet to better describe the association between post COVID‐19 and insomnia. Interestingly, at Jordan University Hospital (JUH) in Amman, Jordan, a case of severe insomnia was reported in which insomnia symptoms started after COVID‐19 and lasted for 6 months which led the patient to visit JUH.

## 2 CASE PRESENTATION

RA is a 41‐year‐old female patient, a pharmacist, and a mother of four kids. She is a nonsmoker and does not consume any alcohol. RA is allergic to NSAIDs.

She had COVID‐19 in November 2020 and was treated with oxygen therapy at home. She presented to the outpatient clinic in May 2021 complaining of persistent severe insomnia for 6 months after COVID‐19 infection.

### 2.1 Chief complaint

The patient had insomnia for 6 months which exacerbated in the last 2 weeks (almost inconsistent 2 h per day of sleep without bromazepam), headache (occipital radiating to the frontal area, more in the right), occasional slurred speech and nausea (these attacks occurred once daily and resolved spontaneously), and fibromyalgia exacerbation, including burning sensation of the spine that affects her daily performance and sleeping. Her pain was stronger and stiffer at mornings to the extent that she sometimes needed help getting out of the bed. Also, she complained of pain in the shoulder, spine, and hip at multiple trigger points. In addition to chronic and dry cough associated with shortness of breath (SOB) and chest pain, general fatigue, low appetite, and anhedonia. Her relationship with her daughters was affected too, and she felt detached from them. She even had death thoughts.

### 2.2 Physical examination

Upon physical evaluation, the patient was conscious, oriented and alert. She weighed 78 kg and her height was 169 cm. Her body mass index was 27.3 kg/cm^2^. In addition, she was calm and cooperative but she had a low tone of voice and talked slowly. Although she had a depressed mood, her thoughts were clear, logical, concentrated and goal‐directed without perceptual disturbances.

She did not have any fever and her vital signs were normal and stable, with intact reflexes and cranial nerves. She was not cooperative in performing power test but intact power performance and positive pain pressure points over all joints were performed.

### 2.3 Pre‐COVID‐19

Before being infected with the coronavirus, the patient was a socially active person and an athlete. She was humorous and outgoing. She also had regular sleep schedule with an excellent memory. In 2013, when RA was pregnant, she was diagnosed with migraine and fibromyalgia. She was on pregabalin 75 mg once daily for 3–4 months. Her muscle pain had improved completely since then. She is also an asthmatic patient on inhalers and nebulizers once needed.

### 2.4 Characteristics of the patient's COVID‐19 infection

At the beginning of RA's COVID‐19 disease, she complained of a severe headache with burning sensation (occipital radiating to the frontal area; more painful on the right side). Her pain score, according to the patient, was 10. She also reported that she had auditory hallucinations about voices of wolves for 3 weeks. Additionally, RA had developed severe back pain along her spine, and she was treated by a rheumatologist who prescribed her the following medication: pregabalin 75 mg twice daily, etoricoxib 120 mg once daily, duloxetine 30 mg once daily, and a one shot of dexamethasone 8 mg intramuscular injection. Ten days later, she started to complain of mild respiratory symptoms including nasal congestion, runny nose, anosmia, and ageusia. Symptoms were later exacerbated to severe SOB; her O_2_ sat was 84%–90%. Her pulmonologist performed a chest X‐ray. The report revealed bilateral infiltrates. She was treated with dexamethasone 8 mg intramuscular injection for 2 days, prednisolone syrup, azithromycin 500 mg for 6 days, bilastine, montelukast, inhaled corticosteroids, enoxaparin 6000 IU for 10 days, and oxygen therapy for 2 months. Additionally, after 2 weeks of the mentioned respiratory symptoms, she started experiencing GI symptoms, including severe diarrhea, vomiting, loss of appetite, and blood in stools. She had lost 10 kg but was tested negative for *Helicobacter pylori* and amoebae. After 2 weeks of these GI symptoms, she started to experience severe memory impairment; she was not oriented to place nor time especially after waking up at mornings. She felt detached emotionally from her family members.

### 2.5 Characteristics of the patient's insomnia

The patient has reported that since she was infected with SARS‐CoV‐2, she had inconsistent sleep patterns with only 30–120 min of sleep per day. She tried different sleep medications, including diazepam 10 mg and flupentixol/melitracen. None was beneficial. She also reported that she had difficulty sleeping due to muscle and joint pain. She was presented to a neurologist who prescribed her mirtazapine. It helped her fall asleep but was associated with severe agitation and uncontrolled nervousness. RA had violent behavior toward her children; she was still on pregabalin, etoricoxib, and duloxetine.

## 3 MANAGEMENT

### 3.1 Medications prescribed at JUH clinic

At first, RA was prescribed melatonin 2 mg seven tablets before bedtime for her insomnia. After no noticeable improvement, she was prescribed bromazepam 3 mg two tablets once daily. It increased her sleep duration to 2–4 h per day inconsistently. She was also prescribed citalopram and continued on pregabalin 150 mg once daily.

### 3.2 Hospital course and outcome

The patient was admitted to JUH on August 1, 2021 and discharged on August 4, 2021. During this period, laboratory testing, imaging modalities, sleep study, and specific consultations were done for proper evaluation of her condition, as the following.

#### 3.2.1 Imaging modalities

**FIGURE 1:**
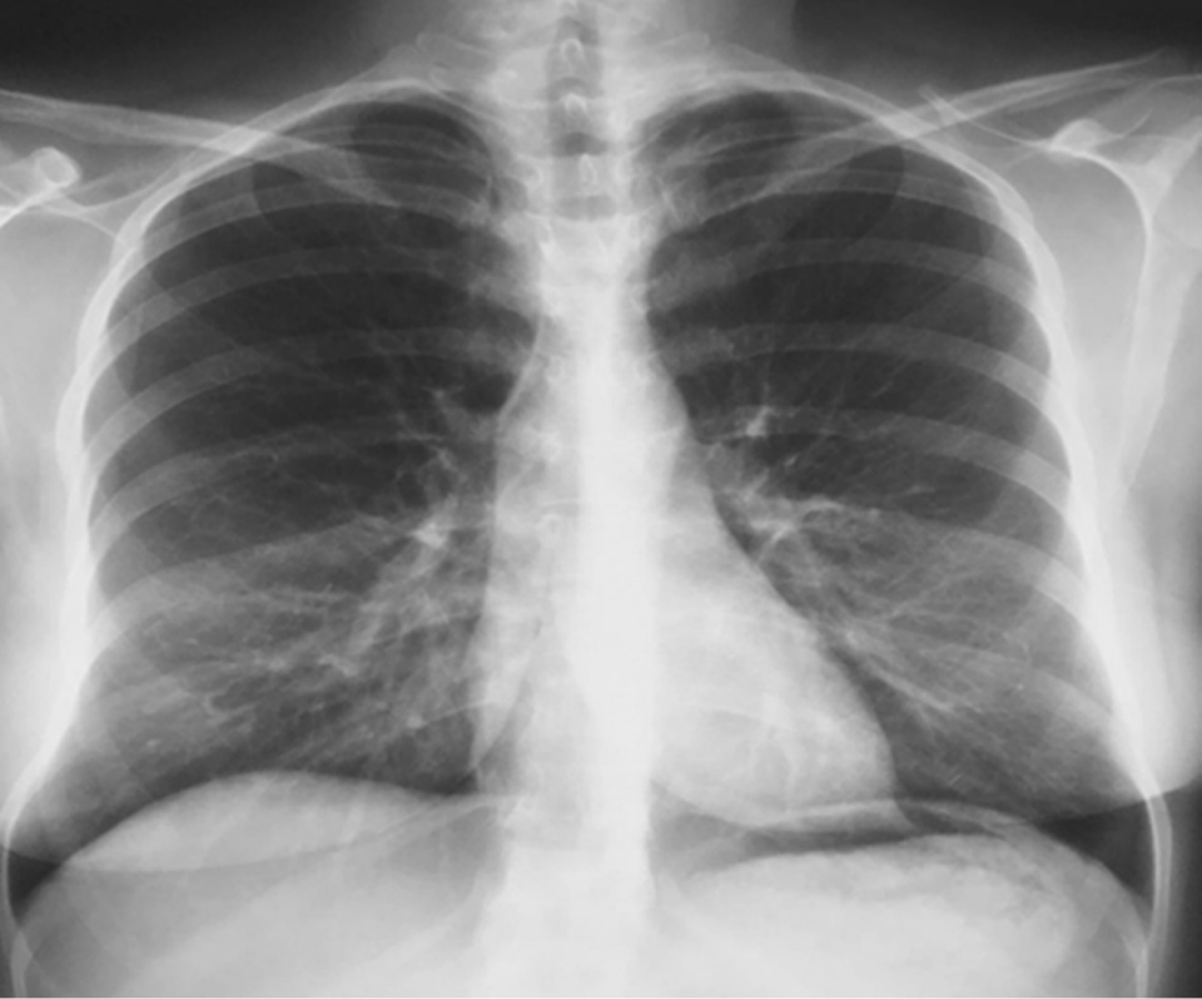
Chest X‐ray for the patient.

**FIGURE 2:**
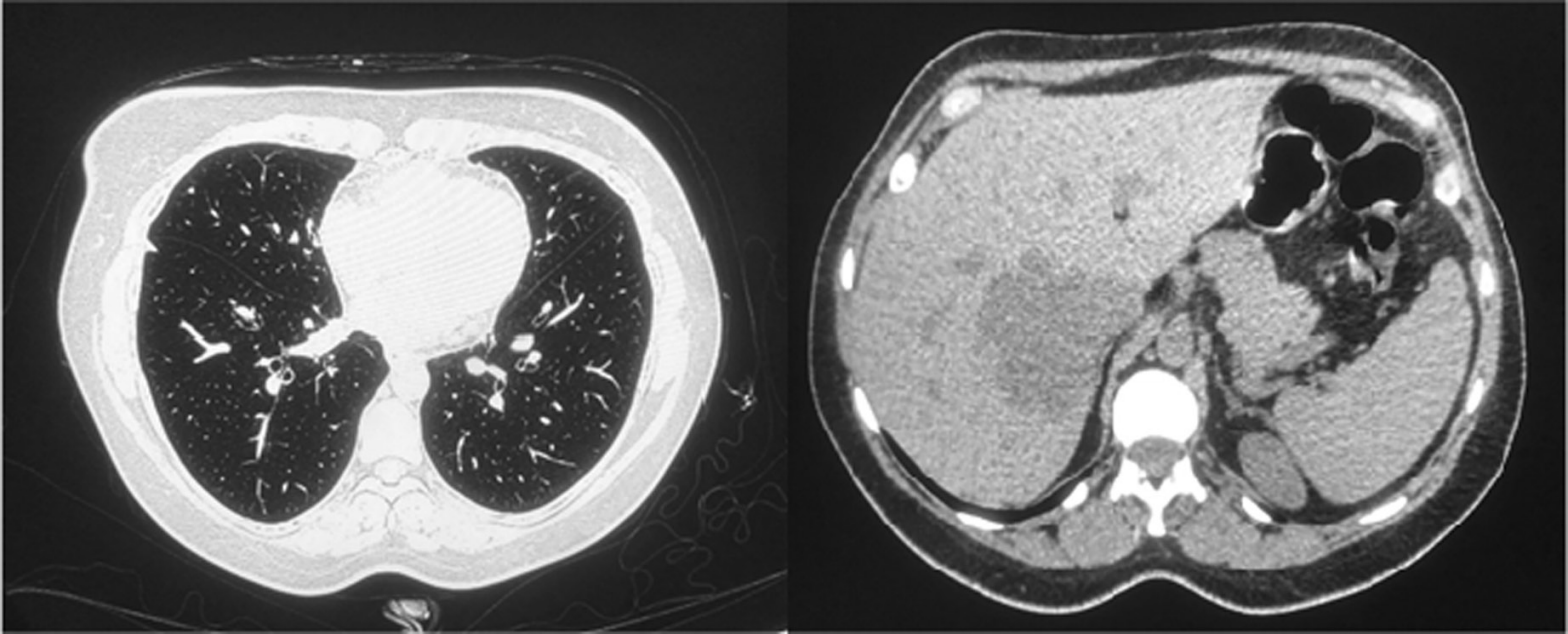
Chest CT scan with contrast for the patient.

**FIGURE 3:**
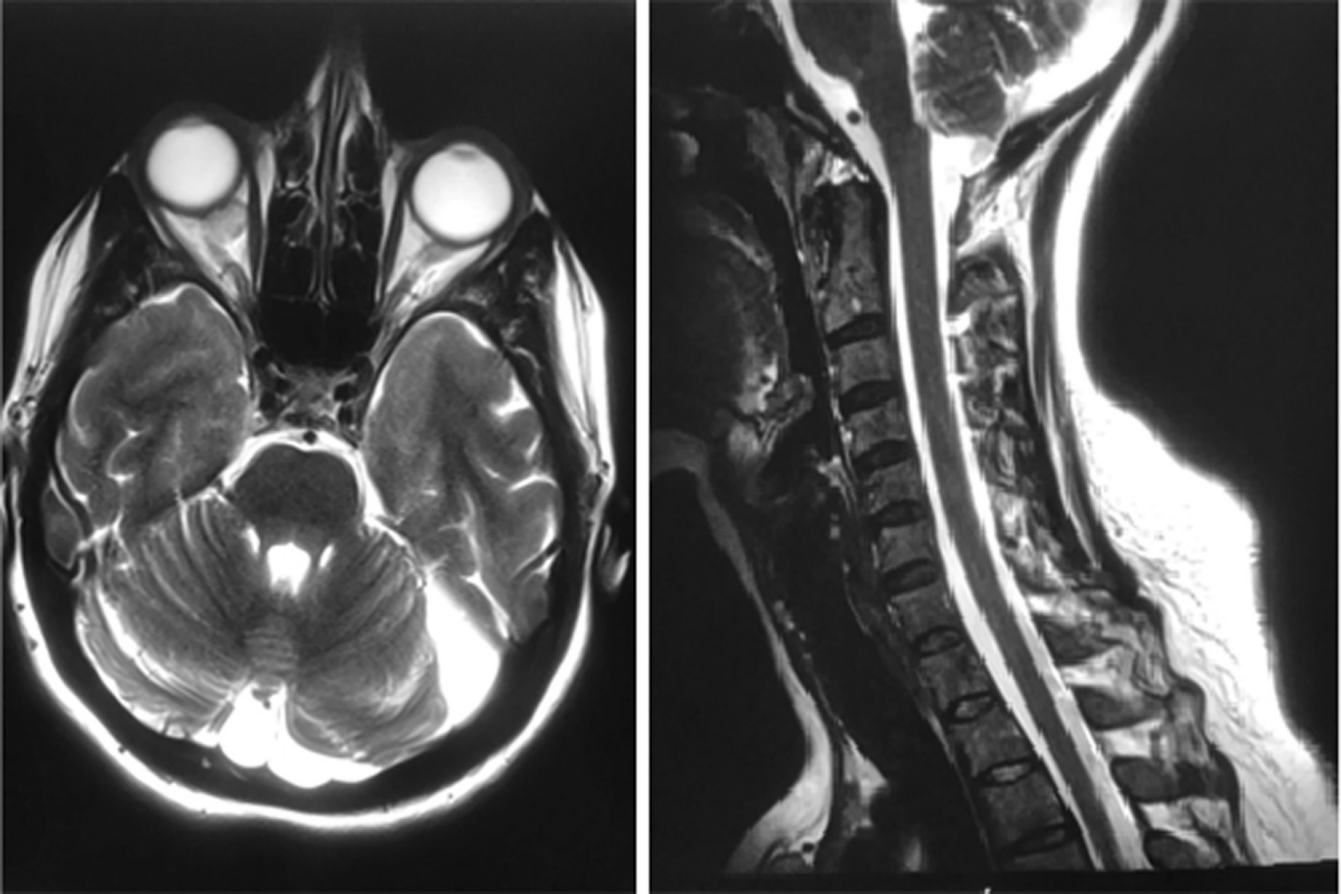
Brain and spine MRI without contrast for the patient.

- *Chest X‐ray*: no infiltrates, no hyperlucency, and appears normal (Figure 1).
- *Chest computed tomography scan with contrast*: three hypodense liver lesions and atelectatic bands. Few small bilateral nonspecific pulmonary nodules, the largest seen at the lateral segment of the right middle lobe measuring 2.5 mm in diameter. No significant intrathoracic lymphadenopathy or pleural effusion (Figure 2).
- *Brain and spine MRI without contrast*: CSF signal intensity lesion was seen on the left side of the posterior fossa associated with hypoplasia of the left cerebellar hemisphere and bony scalloping suggestive of an arachnoid cyst. No abnormal signal intensity, focal lesion in the brain, or hydrocephalus. There was an intraocular right orbital mass indenting the optic nerve sheath complex measuring about 12 mm in maximum dimension. There was a preserved height and alignment of vertebral bodies with no destructive bony lesions, abnormal signals in the spinal cord, or intra‐ or extra‐dural masses. In T5–T6, a small central disk protrusion indenting the thoracic canal was noted, additionally, there was a right posterolateral disk protrusion indenting the right lateral recess in L4–L5. There was a partially included lesion most likely representing a pathologically enlarged left level V lymph node measured 10 mm in the short axis in addition to a few prominent left level III and supraclavicular lymph nodes (Figure 3).

#### 3.2.2 Laboratory results

Upon admission and during the hospital stay, the patient had high C‐reactive protein and erythrocyte sedimentation rate levels. Other laboratory results were mostly normal. Table 1 shows full laboratory results performed at JUH.

**TABLE 1:** Laboratory results performed in the patient on admission, during hospitalization, and at discharge.

Test	Normal range	Results at admission	Results during hospital course	Results at discharge
Random blood sugar, mg/dL	70–110	68	90.7	
Sodium, mmol/L	135–145	140	137	
Potassium, mmol/L	3.5–5.3	4.7	3.93	
Urea, mg/dL	15–46	25	30.7	
Creatinine, mg/dL	0.6–1.2	0.62	0.58	
Chloride, mmol/L	97–110	104	102	
Calcium, mg/dL	8.6–10.2	9.6	8.2	
Phosphorus, mg/dL	2.5–4.5	3.2	3.1	
Magnesium, mg/dL	1.58–2.55	2.06	2.2	
Total bilirubin, mg/dL	Up to 1.1	0.2		
Direct bilirubin, mg/dL	Up to 0.3	0.1		
Total protein, g/dL	6.2–8.7	6.9		
Albumin, g/dL	3.5–5.2	4.2		
Alanine aminotransferase (ALT), U/L	10–35	9		
Aspartate aminotransferase (AST), U/L	13–35	13		
Gamma‐glutamyl transpeptidase (GGT), U/L	5–39	8		
Alkaline phosphatase, U/L	35–129	104		
Hemoglobin, g/dL Hematocrit, percent MCV MCH MCHC RDW	11.6–15 35.5–44.9	11.8 37.9 67.8 21.2 31.3 23.7	11.1 35.2 69.7 22 31.5 22.3	
White blood cells, billion/L Neutrophils% Eosinophils% Basophils% Lymphocytes% Monocytes%	4–10	9.8 73.8% 1.5% 0.5% 19% 5.2%	6.88 58.3% 2.1% 0.6% 32% 7%	
Platelets, billion/L MPV	150–450	269 8.7	285 9.8	
CRP, mg/L	<5	12.3	19.5	
ESR, mm/h	0–20	26	40	
Rheumatoid factor, IU/mL	<14		Negative	
Free T4, pmol/L	11.5–22.7		13.29	
TSH, m IU/L	0.55–4.78		0.72	
Cortisol, nmol/L	94.9–462.4		124.21	
Complement 3 (C3), g/L	0.9–1.8			1.3
Complement 4 (C4), g/L	0.1–0.4			0.4
Extractable nuclear antigen (ENA)	Negative (≤5)			Negative
Antineutrophil cytoplasmic antibodies (ANCA), U/mL	Negative (<10 for both PR3, MPO)			Negative
Antinuclear antibody (ANA)	Negative (<40)			Negative

#### 3.2.3 Sleep study

The patient's Epworth Sleepiness Scale score was 12/24. The sleep efficiency was 44% with a total sleep time of 169 min. Sleep onset was 13 min and rapid eye movement (REM) latency from sleep onset was 239 min with REM time of 29 min. The patient had 31 hypopnea attacks with a sleep distribution of 14% N1, 60% N2, 9% N3, 17% REM, and an overall arousal index of 34 events per hour of sleep. The apnea–hypopnea index was elevated at 11 events per hour of sleep. On the other hand, arterial oxygen saturation (SpaO_2_) was 94% in the awake state and was below 90% for 0.1% of the total sleep time, and finally, snoring time of 34 min (20% of sleep time).

#### 3.2.4 Psychiatry consultation

The patient was diagnosed with major depressive disorder; therefore, she was given bromazepam 3 mg as needed, escitalopram 10 mg once daily, and mirtazapine 30 mg at bedtime.

#### 3.2.5 Neurology consultation

The patient's case was indeterminate and had features suggestive of either fibromyalgia exacerbation or post‐COVID‐19 condition. The neurology team had decided to continue her on pregabalin 150 mg twice daily.

#### 3.2.6 Neurosurgery consultation

Regarding the arachnoid cyst and the orbital mass, contrasted magnetic resonance imaging was decided to be repeated in 6 months.

### 3.3 Discharge

RA was discharged after completing the investigation. She was asked to be adherent on the prescribed medication and to be followed up in the outpatient department.

Unfortunately, the patient was not compliant with her follow‐up appointments and was unreachable by phone; therefore, no further data are available regarding this patient.

## 4 DISCUSSION

In this case report, we presented a confirmed case of COVID‐19. The case mentioned a complaint of sleeplessness for 6 months following COVID‐19. Her condition deteriorated 2 weeks before seeking medical advice. The patient was unable to sleep more than 2 h each night. It improved slightly once she took bromazepam (3 mg). She also reported headaches, slurred speech, nausea, low energy, low appetite, anhedonia, family detachment, and occasional death wishes.

It was critical to conduct sleep studies as soon as possible and take steps to prevent it from worsening. Sleep studies revealed decreased sleep efficiency at 44% with a total sleep time of 169 min, sleep onset was 13 min, and REM latency from sleep onset was 23 min with REM time of 29 min. Furthermore, the arousal index was elevated at 34 events per hour of sleep, 31 hypopnea attacks, awake spaO_2_ was 94%, 0.1% total sleep time spent with spaO_2_ less than 90%, and snoring time was 20% of sleep time.

The majority of COVID‐19‐confirmed patients reported at least one symptom even after 6 months of infection.^6^ Among these stated symptoms, 23% were sleep issues.^6^ Sleep disturbances are fairly prevalent with severe illness and can last up to a year.^7^ To accelerate recovery from COVID‐19 and eliminate the need for emergency medicine, the hazard of serious sickness was dramatically decreased after enhancing sleep quality.^8^ Three in four patients in a case series study reported decreasing quality of sleep, and sleep difficulties, including alterations in sleep latency and daytime performance.^8^

Subjective sleep quality, length, latency, effectiveness, sleep disruption, consumption of hypnotic medication, and daytime functionality were substantially elevated in COVID‐19 survivors.^6^ There have been observations of post‐COVID condition with sleeplessness in persons surviving from COVID‐19, which is similar to the post‐SARS‐COV‐1.^9^ On the contrary, a Chinese study reported that one in four of their targeted sample had sleep disturbance^10^; however, this scale may be underestimated; the authors selected a higher cutoff threshold (>7), resulting in an underestimation of sleep disturbances in the study sample.

The authors of a study of 35 individuals surviving the COVID‐19 discovered a correlation between sleep quality and a specific gender.^11^ In a study conducted by Duan et al. during the pandemic, females were more likely to experience insomnia and anxiety than males, which is significantly different from the sex difference in sleep patterns observed in China before the outbreak time.^(^ ^12^, ^13^ ^)^ Females have a greater frequency of mental problems including posttraumatic stress disorder and anxiety than males, which may be connected to variations in gonadal hormone levels in response to stress and gender disparities in brain areas.^14^

In the present case report, the quality of life (QoL) was decreased with depressed mood, anhedonia, and death wishes. A study conducted by El Sayed et al. reported statistically significant positive connections between the insomnia severity index scale and various domains of the QoL scale, including general well‐being, role constraints due to poor physical health, and role restrictions due to emotional burdens, and overall health.^15^ Another study by Bi and Chen showed that sleeping disturbance is a significant factor leading to depression and poor QoL.^16^

Our case report presented a vital problem for post‐COVID patients, which affected all domains of life and it is not a common complaint to have insomnia for 6 months (2 h/day) after being diagnosed with COVID‐19. The current literature lacks extended follow‐up of insomnia symptoms in COVID‐19 patients. It is worth mentioning that the studied case lacked history of smoking, alcohol, caffeine, and substance use.

We recommend conducting extensive longitudinal studies with a sufficient follow‐up period of confirmed COVID‐19 patients to test the severity of insomnia and its impact on different domains of QoL.

## 5 CONCLUSION

Post‐COVID‐19 condition can affect patients on many aspects, including the psychiatric level. Insomnia can be developed following COVID‐19. Further analysis is required to assess the prevalence and cause of post‐COVID insomnia.

## AUTHOR CONTRIBUTIONS

**Asma Salameh Albtoosh:** Conceptualization; formal analysis; methodology; writing – original draft; writing – review and editing. **Sajeda Ghassan Matar:** Methodology; writing – original draft; writing – review and editing. **Shatha Nizar Bishtawi:** Writing – original draft; writing – review and editing. **Alaa Ahmed Elshanbary:** Methodology; writing – original draft; writing – review and editing. **Lara Ibrahim Ramadan:** Writing – original draft; writing – review and editing. **Andrew Bradbeer:** Writing – original draft; writing – review and editing. **Elfatih A. Hasabo:** Writing – review and editing. **Iman A. Basheti:** Writing – review and editing.

## FUNDING INFORMATION

The study was funded by the authors themselves.

## CONFLICT OF INTEREST STATEMENT

The authors have no conflict of interest to declare.

## ETHICS STATEMENT

Verbal and written consents were obtained from the patient before writing the case or using investigations to participate.

## CONSENT

Written consent to publish this information was obtained from the patient. This patient has not been reported in any other submission by the authors or anyone else.

## Data Availability

The datasets used and/or analyzed during the current study are available from the corresponding author on reasonable request.
